# Correlations of C-Reactive Protein and Folate with Smoking, Sport, Hematological Inflammation Biomarkers and Anthropometrics in Syrian University Female Students Cross-Sectional Study

**DOI:** 10.1038/s41598-019-51658-z

**Published:** 2019-10-23

**Authors:** Mazen Rajab, Mohamad Jumaa, Muhammad Yusr Horaniah, Ahmad Barakat, Saied Bakleh, Wafika Zarzour

**Affiliations:** Biochemistry Department, Faculty of Pharmacy, Arab International University, Damascus, Syria

**Keywords:** Predictive markers, Body mass index, Risk factors

## Abstract

In Syria, high-Sensitive C-Reactive (hsCRP), folate, and, other health risk data in young women are limited. This cross-sectional study evaluates hsCRP and folate levels along with anthropometric characteristics, lifestyle factors and some biomarkers linked to cardiovascular disease (CVD) risk factors in healthy female students (n = 207, 18–25 years old). Among participants, hsCRP level was at average or high risk of CVD in 20.7% and 2.5% respectively and it was significantly higher in participants who had high body mass index (BMI) (Nonparametric statistical tests, *p* value < 0.05). Unexpectedly, it did not vary significantly between smokers and nonsmokers. And, it correlated positively with anthropometric and erythrocyte sedimentation rate (ESR) measurements. While folate level was low in 3.4% of participants, no association between hsCRP and folate levels was found. Finally, low hemoglobin level and habit of waterpipe smoking are spreading; and, sport practicing is shrinking. After reviewing similar works, this study suggests that the possible correlation between hsCRP and folate could be displayed in patients older than 30 years. Also, the marked decrease in hemoglobin level needs more attention. Finally, young females in Syria are advised to consider a lifestyle free of smoking and packed with physical activity.

## Introduction

Folate is one of the B vitamins needed to produce and maintain new cells. And it is required for the formation of heme of the hemoglobin in erythrocytes (red blood cells). Furthermore, it lowers the blood’s concentration of homocysteine by playing a methyl donor to remethylate homocysteine to methionine^1^. The homocysteine is a byproduct of consuming meat^2^ and a well known factor, biomarker, for the development of atherosclerosis or heart disease^3^. Hence folate administration may delay the formation of lesions resulted from atherosclerosis^4^ which is the hardening of arteries process caused by the deposition of plaques of fatty material, cellular waste products, calcium, and, other substances on the inner walls of medium or large arteries.

The aforementioned proposed mechanism, among others, is behind the debate on the preventive role of folate in CVD. Therefore, several studies investigated the correlation between levels of folate and C-reactive protein (CRP) which is a blood indicator for inflammation in the body, and when it is measured by high-sensitive method (hsCRP)^5–10^, it is considered a valuable CVD biomarker in young adults^11,12^.

Even though CVD risk factors tend to track from childhood into adulthood^13^, lots of studies on CVD risk ignore young people. Particularly in Syria, where there is a need to examine the lifestyle factors, biological indicators, and, biomarkers associated to CVD risk.

Recently, our team studied some of the CVD risk factors, including hsCRP, in male students aged between 18 and 25 years^14^. Previously, we were interested in female students with the same range of ages to observe the association between Mediterranean diet patterns along with some of CVD risk factors, excluding hsCRP^15^. This age category has equal numbers of males and females, and, presents around 17% of the population in Syria^16^.

Considering the importance of folate in females at childbearing age and to examine the aforementioned effect of folate using hsCRP as a CVD risk marker in healthy young females, our aim of the present work was to investigate, in female students (18–25 years), the folate and hsCRP levels and their correlations with anthropometric measurements, life style, hemoglobin, some of the hematological inflammation biomarkers such as erythrocyte sedimentation rate (ESR) and hematocrit (HCT)^17^, and, some blood components that may interfere in inflammation process such as white blood cells (WBC) and platelets^18^. Also, mean red cell volume (MCV), mean red cell hemoglobin (MCH), and, mean red cell hemoglobin concentration (MCHC) were investigated because they are calculated using HCT measurement, hemoglobin concentration, and, red blood cells (RBC) counts. In addition, we discussed similar studies, and compared them with our present work.

## Materials and Methods

All methods were carried out in accordance with guidelines and regulations provided by Syrian ministry of health, which is the medicines regulatory authority that accredits medical laboratories. The research laboratory of Arab International University (AIU) and Alkhatib laboratory are licensed by ministry of health decision number 8081 date December 18, 2008 and decision number 7367 date December 14, 1993, respectively. Also, the research was done after obtaining the approval of AIU presented by Faculty of Pharmacy Board (resolution: No. 3/16, April 5, 2017) and Postgraduate Studies and Research Council (resolution No. 6/5, May 24, 2017). All participants provided written informed consent.

### Participants

Between 1 and 20 June 2018, the research team explained the aim of the study to seven hundred young female students in the faculty of pharmacy at AIU. Two hundred and seven (𝑛 = 207) healthy females agreed to participate in the study and matched the following criteria: Non-pregnant women aged between 18 and 25 had neither acute nor chronic medical history (diagnosed CVD, cerebrovascular diseases, dyslipidemia, stable hypertension treated by drugs, chronic hepatic disease, and renal problems), and, were not on any kind of either medications or dietary supplement during the past two months.

### Anthropometric, clinical, and lifestyle characteristics

Research team measured participant waist and hip circumference using a measuring tape, and, height using a wall mounted height rod; measurements were approximated to the nearest 0.5 cm. Weight was measured, to the nearest 100 g, by a calibrated scale (Health Scale RGT-160, manufactured by Salter Brecknell^19^). Body mass index (BMI, kg/m^2^) was calculated by dividing weight (kg) by the square of the standing height (m^2^), and waist-to-hip ratio (WHR) resulted from dividing waist circumference by hip circumference.

World health organization (WHO) and National Heart, Lung and Blood Institute (NHLBI) considered waist circumference as an indicator of health risk associated with excess fat around the waist. Thus, waist circumference greater than 88 cm in women is associated with health problems such as type 2 diabetes, CVD, hypertension, and dyslipidemia^20–22^.

Also, WHO standards^20^ state that abdominal obesity for females is defined as a WHR is equal to or more than 0.85, or, a BMI more than 30 kg/m^2^. Hence, results of waist circumference and WHR were classified by their risks of disease and cross tabulated with BMI obesity classification.

Structured interview method using a questionnaire from our previous studies^14,15^ was applied by our research team to reveal the participant’s age, smoking habit, drinking alcohol, physical activities, medications, and personal, parental medical history; however, information about diet was not collected.

The participant’s habits of drinking alcohol, smoking and doing sports were described as rarely, monthly, weekly, and daily. It was noted if a sport needs a club to be practiced or not.

Also, regional traditions permit to distinguish between two types of smoking: cigarettes, and waterpipe (hubble-bubble, shisha, nargile, and hookah have similar structure in which the smoke passes through water, causing a bubbling sound). In addition, the number of smoked cigarettes per day was reported.

Parent medical history investigation showed that 42 participants had at least one of their parents affected with one of the following diseases (type 2 diabetes, CVD, dyslipidemia, and anaemia) but it was difficult to obtain more useful information to our study.

WHO standards and participant’s life style resulted groups were reported with numbers of participant, medians of hsCRP and folate levels.

### Laboratory analyzes

Laboratory analyzes were done at the medical research laboratory of AIU and Alkhatib laboratory. Venipuncture was performed for each participant by applying a latex rubber strap and using a 10 mL syringe. 5 mL of blood was immediately transferred to the first tube to obtain the serum by centrifuge separation, and 2.5 mL to the second tube containing the anticoagulant ethylene diamine tetraacetic tripotassium salt (K_3_EDTA) for blood counting, finally, 2.5 mL to the third tube containing sodium citrate as anticoagulant for ESR measurement.

ESR was measured by the Westergren method^23^. HCT is the ratio of the volume of red blood cells to the total volume of blood. The HCT and complete blood count were performed using an automated hematology analyzer^24^ ABX MICRO 60. Precisions of WBC, RBC, hemoglobin concentration, HCT, and, Platelets were <2.5%, <2%, <1.5%, <2%, <5%, respectively.

MCV which is the volume of the average red blood cell was calculated by multiplying the hematocrit by 10 and dividing by RBC.

MCH which is the average mass of hemoglobin per red blood cell was calculated by dividing the hemoglobin concentration by RBC.

MCHC which is the number of grams of hemoglobin per unit volume of packed red blood cells, was calculated by multiplying hemoglobin concentration by 100 and dividing by the HCT.

hsCRP was measured in serum by particle enhanced immunonephelometry method using Siemens Healthcare CardioPhase® hsCRP on BN ProSpec System^25^ (fully automated bench top analyzer). The manufacturer claims that both lower limit of detection (LOD) and lower limit of quantification (LOQ) are equal to 0.175 mg/L. In this study, the minimum observation of hsCRP (0.22 mg/L) was higher than LOQ.

Folate concentration determination was done by electrochemiluminescence immunoassay (ECLIA) method in serum using Roche Elecsys Modular Analytics E170^26^. The manufacturer claims that lower limit of measurements of Blank (LOB) = 0.6 ng/mL (1.36 nmol/L), LOD = 1.2 ng/mL (2.72 nmol/L), LOQ = 2.0 ng/mL (4.54 nmol/L) with a total allowable relative error of ≤20%. In this study only two observations of folic acid were at 97.5% of LOQ (1.95 ng/mL), in addition, nonparametric statistical methods were used; therefore they were included in the investigation.

Hematological participants’ results were grouped by low, normal, and high levels of hematological age-specific reference ranges established by Department of Pathology, School of Medicine, Virginia Commonwealth University (VCU)^27^.

hsCRP results of participants were grouped by low, average, and high risk levels of American Heart Association (AHA) and US Centers for Disease Control and Prevention recommendations^28^.

### Statistical analysis

In Table 1 and 2, the mean and median were calculated to estimate the central tendency of continuous variables like anthropometric and hematological characteristics of participants.

**Table 1:** Anthropometric characteristics of participants (𝑛 = 207).

Characteristic, unit	Mean ± SD	Median (Q1-Q3)	Minimum	Maximum
Age, year	21.04 ± 1.60^a^	20.93 (19.64–22.28)	18.39	24.78
Height, m	1.63 ± 0.06^a,b^	1.62 (1.58–1.67)	1.46	1.79
Weight, kg	60.81 ± 10.87^a^	58.90 (53.05–66.50)	35.80	110.00
BMI, kg/m^2^	22.94 ± 3.76^c^	22.07 (20.17–24.65)	15.29	42.44
Waist, cm	71.72 ± 8.96^c^	70.00 (65.00–78.00)	52.00	104.00
Hip, cm	96.61 ± 8.57^a^	95.00 (90.00–102.50)	78.00	131.00
WHR, ratio	0.74 ± 0.06^a^	0.74 (0.70–0.78)	0.60	0.94

Note: Q1, first quartile; Q3, third quartile.

^a^Normal distribution was proved by Kolmogorov-Smirnov test (*p* < 0.05).

^b^Normal distribution was proved by Shapiro-Wilk test (*p* < 0.05).

^c^After the elimination of 2 outliers, which were out the range of 2.5^th^ and 97.5^th^ percentiles, the normal distribution was proved using Kolmogorov-Smirnov test (*p* < 0.05). Only mean ± SD were recalculated.

Damascus, Syria, June 2018.

**Table 2:** Hematological characteristics of participants (𝑛 = 207).

Characteristic, Unit	Mean ± SD	Median (Q1–Q3)	Minimum	Maximum
White blood cell count (WBC), 10^9^ cells/L	7.75 ± 1.9^a^	7.6 (6.4–8.8)	3.6	16.5
Red blood cell count (RBC), 10^12^ cells/L	4.79 ± 0.42^a^	4.76 (4.56–4.99)	3.98	7.72
Hemoglobin (HB), g/dL	11.89 ± 0.92^a,b^	11.9 (11.3–12.5)	9.4	18.2
Hematocrit (HCT), %	37.38 ± 2.52^a,b^	37.3 (35.55–39.1)	29.8	60.1
Mean red cell volume (MCV), fL/cell	78.35 ± 5.74^a^	79 (76–82)	58	89
Mean red cell hemoglobin (MCH), pg/cell	24.94 ± 2.27^c^	25.5 (23.7–26.4)	17.1	30.5
Mean red cell hemoglobin conc. (MCHC), g/dL	31.76 ± 0.77^d^	31.9 (31.4–32.3)	3.1	36.1
Platelet, 10^9^ cells/L	282.86 ± 59.24^d^	269 (235–321.5)	110	517
Erythrocyte sedimentation rate (ESR), mm in 1 hr	19.00 ± 15.05^c^	15 (7–27)	1	72
High sensitive CRP (hsCRP), mg/L	0.86 ± 1.15^c^	0.52 (0.36–0.94)	0.22	12.85
Folate, ng/mL	7.24 ± 3.26^c^	6.67 (5.01–8.61)	1.95	16.91

Note: Q1, first quartile; Q3, third quartile.

^a^Normal distribution was proved by Kolmogorov-Smirnov test (*p* < 0.05).

^b^After the elimination of 2 outliers, which were out the range of 2.5^th^ and 97.5^th^ percentiles, the normal distribution was proved using Shapiro-Wilk test (*p* < 0.05). Only mean ± SD were recalculated.

^c^No normal distribution, mean ± SD was cited without any further interpretation.

^d^After the elimination of 2 outliers, which were out the range of 2.5^th^ and 97.5^th^ percentiles, the normal distribution was proved using Kolmogorov-Smirnov test (*p* < 0.05). Only mean ± SD were recalculated.

Damascus, Syria, June 2018.

Standard deviation (SD), minimum, maximum, and first and third quartiles values (Q1-Q3) were calculated to describe the dispersion of these variables. Q1 and Q3 were boundaries of the middle half of the set of data.

Kolmogorov-Smirnov (KS) and Shapiro-Wilk (SW) tests were used to assess the normal distribution for each variable. Statistical significance was defined at *p* < 0.05.

KS test is widely used and it is a discrepancy measurement based on the largest vertical difference calculated between cumulative distributions of normal distribution and empirical distributions function which is estimated based on data. In contrast, SW test is less frequent and it is a correlation measurement based on the ratio of two estimates of scale obtained from order statistics. The two estimates are normally distributed weighted least squares estimate (estimated variance) and the data variance. SW test is too sensitive and could have problem if many equal values are present in data. Nevertheless, among four tests of normality SW, KS, Lilliefors, and Anderson-Darling, SW is the most powerful test whereas KS test is the least powerful test^29^.

To note any significance of differences between groups, the nonparametric Wilcoxon rank sum test (WRST) for independent samples was performed.

To detect the effects of BMI, waist circumference, and WHR on hsCRP and folate levels; a table (Table 3) was constructed to show the groups classified by WHO and NHLBI standards^20–22^ and to report median levels of hsCRP and folate with the number of participants in each classified group. WRST was used to show significance difference in hsCRP and folate levels between each group and the overall group.

**Table 3:** Participant’s anthropometric measurements grouped by BMI, waist circumference, and WHR^a^.

BMI (kg/m^2^), class	Median of hsCRP mg/L, Folate ng/mL (𝑛) (Disease risk)				
	Waist circumference			WHR	
	Totals	≤88 cm	>88 cm	<0.85	≥0.85^b^
Overall	0.52, 6.67 (207)	0.50, 6.66 (196)	1.88, 8.95 (11)	0.52, 6.66 (198)	0.81, 7.04 (9)
<18.5, underweight	0.37, 7.02 (13)	0.37^**^, 7.02 (13) (Non)	– (0) (§)	0.37, 7.02 (13)	– (0)
18.5–24.9, normal	0.46, 6.74 (146)	0.46, 6.70 (144) (Non)	1.22, 8.39 (2) (§)	0.46, 6.73 (141)	0.38, 6.76 (5)
25.0–29.9, overweight	0.77^*^, 6.32 (34)	0.7^**^, 6.11 (32) (Increased)	1.88, 8.72 (2) (High)	0.71^*^, 6.11 (32)	1.11, 7.77 (2)
30.0–34.9, obese I	0.96, 5.17 (11)	0.90, 4.80 (7) (High)	1.99, 7.77 (4) (Very high)	0.95, 5.42 (10)	1.88, 5.17 (1)
35.0–39.9, obese II	1.50, 9.93 (2)	– (0) (Very high)	1.50, 9.93 (2) (Very high)	1.55, 9.56 (1)	1.45, 10.29 (1)
40 or greater, obese III	5.63, 3.43 (1)	– (0) (Extremely high)	5.63, 3.43 (1) (Extremely high)	5.63, 3.43 (1)	– (0)

Note: BMI, body mass index; hsCRP, high-sensitive C-reactive protein;-, empty group; §, increased waist circumference also can be a marker for increased risk, even in persons of normal weight.

^a^Median of hsCRP (mg/L), Median of folate (ng/mL) levels and participant numbers (𝑛) of each group were reported with disease risk for type 2 diabetes, hypertension, and CVD relative to normal weight and waist circumference of BMI and waist circumference^20–22^. Wilcoxon rank sum test was used to show significance difference between each group of participants and the overall group.

^b^(Increased risk).

^*^0.001 ≤ *p* value < 0.025.

^**^0.025 ≤ *p* value < 0.05.

The overall group (𝑛 = 207). Damascus, Syria, June 2018.

To detect the habit effects of alcohol drinking, smoking, or sport on hsCRP and folate levels; a table (Table 4) was constructed to report median levels of hsCRP and folate with number of participants in each group formed by habit frequency (or cigarettes number per day in case of cigarette smoking). WRST was applied with similar manner and reason to the previous.

**Table 4:** Participants grouped by alcohol drinking, smoking, or sport habits characteristics^a^.

Habit	Median of hsCRP, mg/L, Median of folate ng/mL (*n*) Habit frequencies					
	No	Yes	Rarely	Monthly	Weekly	Daily
Alcohol drinking	0.52, 6.68 (194)	0.52, 6.63 (13)	0.61, 6.50 (4)	0.78, 6.63 (5)	0.42, 3.80 (3)	0.22, 7.37 (1)
Sport	0.52, 6.74 (95)	0.48, 6.67 (112)	0.65, 8.12 (9)	0.52, 5.70^*^ (31)	0.48, 6.73 (47)	0.44, 6.91 (25)
No club		0.46, 6.68 (63)	0.80, 7.78 (8)	0.48, 5.85 (20)	0.40, 6.97 (22)	0.46, 6.91 (13)
Club		0.52, 6.31 (49)	0.35, 8.12 (1)	0.57, 5.26 (11)	0.66, 5.75 (25)	0.44, 6.85 (12)
Smoking	0.52, 6.75 (113)	0.52, 6.24 (94)	0.40, 6.96 (6)	0.58, 6.67 (30)	0.61, 7.03 (34)	0.50, 5.75^*^ (27)
Smoking by tobacco type						
Waterpipe^b^	0.52, 6.74 (119)	0.52, 6.24 (88)	0.36, 6.87 (5)	0.61, 6.38 (28)	0.60, 7.02 (29)	0.48, 5.71^*^ (26)
Number of cigarettes per day (𝑥)			0 < 𝑥 ≤ 5	5 < 𝑥 ≤ 10	10 < 𝑥 ≤ 20	𝑥 > 20
Cigarettes	0.50, 6.67 (195)	0.59, 6.85 (12)	2.05, 9.11 (1)	0.41, 7.02 (4)	0.63, 7.04 (5)	0.89, 6.03 (2)
Both	0.52, 6.66 (201)	1.03, 7.31 (6)	– (0)	1.15, 5.67 (2)	– (0)	1.25, 6.16 (1)

Note: hsCRP, high-sensitive C-reactive protein.

^a^Median of hsCRP (mg/L), and folate (ng/mL) with participant numbers (*n*) of each group were shown. Wilcoxon rank sum test was used to show significance difference between each group of participants and the overall group (*n* = 207)

^b^Hubble-bubble, shisha, nargile, and hookah have similar structures in which the smoke passes through water, causing a bubbling sound.

^*^p value = 0.07 only in three groups; in general the significance level was more than 0.20.

The overall group (*n* = 207). Damascus, Syria, June 2018.

To show the distribution of hematological measurements; a table (Table 5) was constructed to report the median of hematological measurements and number of participants in each group formed by VCU range^27^.

**Table 5:** Participant hematological measurements grouped by low, normal, or high levels of hematological reference values of VCU.

Characteristic, Unit	[VCU Normal range]	Median (𝑛)		
		Low	Normal	High
White blood cell count (WBC), 10^9^ cells/L	[3.9–11.7]	3.60 (1)	7.60 (200)	12.70 (6)
Red blood cell count (RBC), 10^12^ cells/L	[3.85–5.16]	– (0)	4.71 (181)	5.39 (26)
Hemoglobin (HB), g/dL	[12.0–15.0]	11.30 (108)	12.50 (98)	18.20 (1)
Hematocrit (HCT), %	[34.8–45.0]	33.60 (31)	37.90 (174)	52.70 (2)
Mean red cell volume (MCV), fL	[78.5–96.4]	74.00 (85)	82.00 (122)	– (0)
Mean red cell hemoglobin (MCH),pg/cell	[25.6–32.2]	24.00 (110)	26.50 (97)	– (0)
Mean red cell hemoglobin conc. (MCHC), g/dL	[30.5–34]	29.90 (17)	32.00 (189)	36.10 (1)
Platelets, 10^9^ cells/L	[172–440]	139.50 (2)	268.00 (202)	445.00 (3)
Erythrocyte sedimentation rate (ESR), mm in 1 hr	[0–20]	§	9.00 (134)	32.00^*^ (73)
Folate, ng/mL	>3.00	2.41 (7)	6.74 (200)	§
hsCRP, mg/L	≤5.00	§	0.52 (204)	6.07 (3)

Note: VCU, hematological age-specific reference ranges established by department of pathology, school of medicine, Virginia Commonwealth University^27^. Wilcoxon rank sum test was used to show significance difference in hsCRP or folate levels between each group of participants and the overall group. 𝑛, number of participants of each group; –, empty group; §, not applicable.

^*^p value = 0.02 and the median of hsCRP was 0.70 mg/L.

The overall group (𝑛 = 207). Damascus, Syria, June 2018.

And to detect the effects of the distribution of hematological measurements on hsCRP and folate levels, WRST was applied similarly to the previous. The hsCRP median of one group was reported in footnote of this table because WRST detected only a significant difference between this group and the overall group.

To examine the relationship between hsCRP and folate levels, a table (Table 6) was constructed to report median levels of hsCRP and folate with number of participants in each group formed by AHA range^28^. WRST was applied similarly to the previous.

**Table 6:** Folate results grouped by AHA of hsCRP. The overall group (𝑛 = 207).

	Low risk (<1 mg/mL)	Average risk ([1–3] mg/mL)	High risk (>3 mg/mL)
Median of hsCRP mg/L, Median of folate ng/mL (𝑛)	0.42, 6.68 (159)	1.34, 6.56 (43)	5.63, 3.52 (5)

Damascus, Syria, June 2018.

Note: AHA, the American Heart Association and U.S. Centers for Disease Control and Prevention recommendations for hsCRP testing and CVD risks^28^. Wilcoxon rank sum test did not show significance difference in hsCRP or folate levels between any group of participants and the overall group; 𝑛, number of participants of each group.

To recognize hsCRP, folate levels and other measurements patterns among participants, principle component analysis could not be applied because the validity of all possible correlation matrices and samples sizes’ (number of participants into different groups) tests showed too little correlation between variables; Bartlett’s test for homogeneity of variance showed *p* > 0.05.

Also, the assumption of normal distribution of prediction errors was not valid because the residuals of the fitted linear regression models were not normally distributed; hence, linear regression models could not be applied. In addition, it may need larger number of participants.

Hence, to examine the effect of BMI, smoking, sport, and the presence of medical history in one of the participants’ parents on the association patterns of hsCRP or folate along with other variables; for each examined factor, participants were divided into two opposite groups using appropriate inclusion criteria as shown in Table 7 which lists group names with the inclusion criteria (group criteria) of each group. To attest the significant difference between the two opposite groups of each examined factor, WRST was applied.

**Table 7:** Using appropriate inclusion criteria, the participants were divided into two opposite groups^a^.

Group name	Group criteria	*n*	Median (Q1–Q3) mg/L		Variable name (Kendall’s tau-b correlation coefficient) associated with:		
			hsCRP	Folate	hsCRP	, or	folate
Overall	No condition	207	0.52 (0.36–0.94)	6.67 (5.01–8.61)	Weight (0.25)^*^, BMI (0.28)^*^, waist (0.30)^*^, hip (0.27)^*^, WHR (0.17)^*^, WBC (0.13)^**^, HCT (−0.10)^***^, ESR (0.18)^*^.		No correlations.
Low BMI	BMI <25 kg/m^2^	162	0.46^#^ (0.35–0.78)	6.76 (5.02–9.31)	Weight (0.13)^**^, BMI (0.17)^**^, waist (0.19)^*^, hip (0.17)^**^, ESR (0.11)^***^.		Hip (0.12)^**^.
High BMI	BMI ≥25 kg/m^2^	45	0.94^#^ (0.57–1.88)	6.13 (4.8–8.12)	Weight (0.23)^***^, BMI (0.29)^**^, waist (0.33)^**^, WHR (0.29)^**^.		Waist (0.21)^***^, MCHC (−0.21)^***^.
Nonsmokers	Nonsmokers	113	0.52 (0.36–0.91)	6.75 (5.21–8.98)	Weight (0.18)^**^, BMI (0.21)^**^, waist (0.26)^*^, HIP (0.19)^**^, WHR (0.16)^**^, WBC (0.19)^**^, ESR (0.22)^**^.		No correlations.
Smokers	Smokers	94	0.52 (0.36–1.12)	6.24 (4.87–7.86)	Weight (0.32)^*^, BMI (0.34)^*^, waist (0.36)^*^, hip (0.35)^*^, WHR (0.20)^**^.		No correlations.
Frequent sport	Weekly or more frequent	103	0.48 (0.35–0.91)	6.66 (5.06–7.79)	Weight (0.26)^*^, BMI (0.25)^*^, waist (0.29)^*^, hip (0.30)^*^.		No correlations.
No sport	Monthly or less frequent	104	0.54 (0.36–1.05)	6.77 (4.83–9.75)	Weight (0.24)^*^, BMI (0.28)^*^, waist (0.30)^*^, hip (0.24)^*^, WHR (0.21)^**^, WBC (0.17)^**^, ESR 1 h(0.23)^**^		No correlations.
Parents without medical history	No parents medical history for participant	165	0.52 (0.36–0.93)	6.67 (5.06–8.74)	Weight (0.21)^*^, BMI (0.24)^*^, waist (0.26)^*^, hip (0.22)^*^, WHR (0.17)^**^, WBC (0.13)^**^, HCT (−0.11)^***^, ESR (0.22)^*^.		No correlations.
Parents with medical history	At least one parent had medical history	42	0.52 (0.36–0.99)	6.68 (4.37–8.33)	Weight (0.44)^*^, BMI (0.40)^*^, waist (0.47)^*^, hip (0.52)^*^.		RBC (0.26)^**^, MCH (−0.22)^***^, MCHC (−0.25)^**^.

Note: *n*, Number of participants in the group; Q1, first quartile; Q3, third quartile.

^a^To check the significance of difference between them, in their hsCRP (mg/L) or folate (ng/mL) levels, except for the group that contains all participants, Wilcoxon rank sum test of significance difference was applied, separately, on hsCRP, and folate levels between every two successive groups. To reveal the possible associations, Kendall’s tau-b correlation coefficients between variables were calculated.

^*^p value < 0.001

^**^0.001 ≤ *p* value < 0.025.

^***^0.025 ≤ *p* value < 0.05^.^

^#^*p* value < 0.001 of Wilcoxon rank sum test of significance difference between these two marked groups; other *p* values of Wilcoxon test between opposite groups were more than 0.137.

And to study the association patterns of hsCRP or folate levels with other variables without the assumption of binormality between two variables or any particular distribution, a nonparametric correlation coefficient was needed. The widely used Kendall’s tau-b coefficient is easier to understand, interpret, and explain than other correlation coefficients. Kendall’s tau-b correlation coefficients between hsCRP or folate levels and other variables were calculated and hypothesis testing for Kendall’s tau-b using normal approximation was used to evaluate the importance of correlation coefficient values.

**Figure 1:**
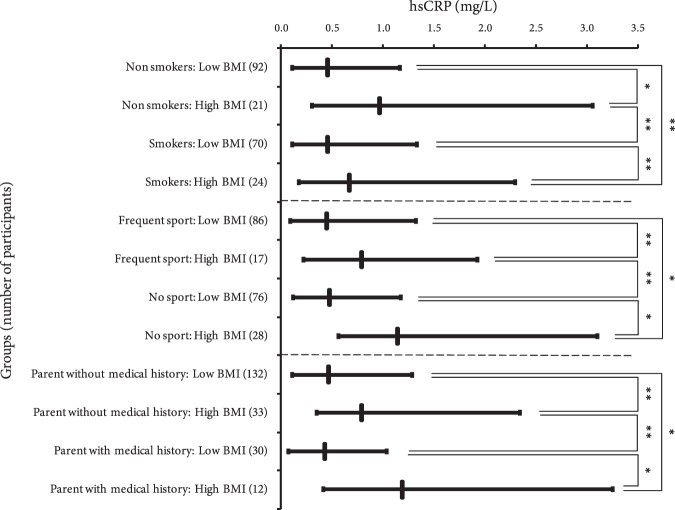
To investigate the effect of the participant’s BMI along with smoking, sports practicing, and the presence of medical history in one of the participant’s parents on hsCRP levels, each group of participants was separated, according to their BMI levels, into two groups. Vertical lines denote the median; horizontal lines represent participant’s group enclosed in interquartile range. Wilcoxon rank sum test of significance difference (*p* value) was applied between different pairs of groups. *p value < 0.001. **0.001 ≤ *p* value < 0.025. Note: hsCRP, high-sensetive C-reactive protein. There was no *p* value between 0.025 and 0.05.

Finally, to investigate the joint effects of participants’ BMI with smoking, sports practicing, or the presence of medical history in one of the participants’ parents on hsCRP or folate levels; each group of participants listed in Table 7, except for BMI groups, was divided according to BMI of participants into low (BMI < 25 kg/m^2^) and high (BMI ≥ 25) groups. The resulted groups were presented in Fig. 1. WRST was applied on any possible pair of groups formed from the four groups of each investigated factor. In general, statistical significance was defined at *p* < 0.05. Excel software 2007 (Microsoft) was used for the statistics calculations.

## Results

Tables 1 and 2 show anthropometric measurements, and results of laboratory analyzes, respectively. Roughly, all variables had normal distribution except for ESR, hsCRP, and folate results which were skewed to low levels and MCH was twisted to high levels. The hsCRP and folate medians of overall group were 0.52 mg/L and 6.67 ng/mL, respectively.

Table 3 shows that hsCRP and folate were normal in approximately 95% of 207 participants who had normal waist circumference or normal WHR. Nearly, 6% of participants are under weight with normal circumference and WHR, they also had hsCRP median (0.37 mg/L) significantly lower than the median of overall group.

Around 70% of participants had normal weight and waist circumference; among them only 3.4% had WHR equal to or bigger than 0.85 cm.

Roughly 16% of participants were overweight and most of them had normal waist circumference and WHR; their hsCRP median (0.70–0.77 mg/L) was significantly higher than the median of overall group but still below 1 mg/L which makes them at low risk of CVD^28^. Nevertheless WHO and NHLBI classify them at increased disease risk for type 2 diabetes, hypertension, and CVD relative to normal weight and waist circumference^20–22^.

About 7% of participants were affected by obesity (I, II and III) with a hsCRP median (around 1 mg/mL) significantly higher than the median of overall group, in addition 47% of them had waist circumference bigger than 88 cm with a hsCRP median around 2 mg/L.

The median of folate levels did not vary significantly with BMI, waist circumference or WHR groups proposed by NHLBI and WHO^20–22^.

Table 4 shows that only 6.3% of participants are alcohol drinking with hsCRP and folate medians similar to their correspondences in the overall group,

54% of participants practice sport; among of which 27.7% practice sport monthly and had folate median 5.7 ng/mL lower than the folate median of the overall group with a significance p value equals to 0.07.

45.4% of participants are smokers; among of which 28.7% are daily smokers; except one, all of them are waterpipe smokers who had folate median, 5.71 ng/mL, lower than the folate median of overall group with a significance p value equals to 0.07.

Table 5 shows that 2.9%, 12.6%, and 35.3% of participants had levels above the normal range of WBC, RBC and ESR, respectively, and nearly none of participants had low levels of these biomarkers. Only median of hsCRP (0.70 mg/L) for the high ESR group was significantly higher than the median of the overall group.

In more than half of participants, hemoglobin concentration and MCH, separately, were in low range in 52% and 74% of participants, respectively. But considering 11.5 g/dL as the lower limit of normal range of hemoglobin decrease the percentage of low hemoglobin to 31%.

In fact (results not shown in the table), hemoglobin concentration levels were below the normal range when HCT, MCV, MCH, or, MCHC were below their normal ranges in 14.5%, 31.4%, 36.7%, or, 7.7% of participants, respectively.

Apart from the reference ranges, Kendall’s tau-b correlation test shows that hemoglobin concentrations correlate positively with RBC, HCT, MCV, MCH, MCHC, and sport frequency; but correlate negatively with WHR, platelet, and ESR (p < 0.05).

MCV and MCHC were low in 41% and 8.2% of participants, respectively. In addition, all participants with low MCHC had also low MCV.

As shown in Tables 6, 20.7% and 2.4% of participants had a hsCRP level at average and high risk of CVD, respectively. While folate level was low in 3.4% of participants, no association between hsCRP level reported by AHA and folate level was found.

In order to study the effect of BMI, smoking, sport, and participant’s parent medical history, on hsCRP and folate levels, participants were grouped in opposite groups using appropriate criteria.

Hence, Table 7 shows that hsCRP levels were significantly higher in high BMI group compared to their counterparts.

Surprisingly, hsCRP levels were not significantly different between smokers and nonsmokers.

Also, significant positive correlations for hsCRP levels along with all anthropometric measurements were present in all groups except for the correlation with WHR measurements which was absent in low BMI, frequent sport, and parents with medical history groups.

In the overall group, hsCRP levels correlated positively with WBC and ESR levels; and negatively with HCT levels.

Independently, ESR and WBC measurements positively correlated with hsCRP levels in the overall, nonsmokers, no sport, and parents without medical history groups. In addition, ESR levels positively correlated with hsCRP in low BMI group.

HCT levels negatively correlated with hsCRP levels in the overall and parents without medical history groups.

The folate level positively correlated with hip measurements in low BMI group; and in the opposite group it was positively correlated with waist measurements.

MCHC measurements negatively and separately correlated with hsCRP levels first in high BMI group and second in participants who had parent(s) with medical history group.

It is worthy to note (results not shown in the table) that in participants who had parent(s) with medical history, the RBC measurements did not correlate with hemoglobin but they were positively correlated with HCT and folate measurements, and negatively with MCV, MCH, MCHC, and ESR measurements. Also, in the overall group, hemoglobin was correlated with sport practicing significantly.

As Fig. 1 shows, only BMI levels influenced hsCRP levels significantly. It is worth mentioning that folate did not influenced by any factors (results not shown).

## Discussion

The present study was conducted on a group of apparently healthy females, and revealed that hsCRP level of high BMI participants was significantly higher than the level of low BMI group, but still below the average risk of CVD^28^. Even though most of high BMI participants had normal waist circumference and normal WHR; WHO and NHLBI classify them at increased or high disease risk for type 2 diabetes, hypertension, and CVD relative to normal weight^20–22^.

Similar to our preceding study^14^, the correlations of hsCRP along with anthropometric measurements, physical activity, and, hematological biomarkers were present; such as the significant influence of BMI on hsCRP level, and the absence of the effect of smoking on hsCRP level; however, the present study shows that sport habits did not affect the hsCRP level and 23.2% of female participants had a hsCRP level at average or high risk of CVD which was insignificantly less than the 31.7% of male participants reported in our previous study^14^.

The folate level was slightly distinctive in monthly practicing sport and daily smoking groups; nevertheless it was in normal range. While folate level did not vary significantly with BMI, waist circumference, WHR, smoking, sport or participants’ parent(s) medical history groups, correlations of folate level with hip or waist measurements were revealed under opposite conditions of BMI level; which could be explained, first, by the difference of gut microbiota composition between participants in the opposite groups of BMI level reported recently in 2019^30^, and, secondly, by considering that the estimated percentage of recommended dietary allowance^31^ (RDA) of folate that could be provided by the human gut microbiota is up to 37%^32^. Hence, folate level correlation with hip or waist measurements could be a reflection for the activity level of two different compositions of gut microbiota. Nevertheless, in our present study, we could not exclude explanations based on other factors, such as the presence of some specific variants of genes involved in folate metabolism^33,34^ or the differences in folate level in diet pattern.

Furthermore, in participants who had parent(s) with medical history, the folate level correlated positively with RBC, and negatively with MCH and MCHC measurements. Oddly and after considering the role of folate in red bloods cells production, the correlation between folate and RBC was not present in the opposite group. In addition, folate level did not correlate along with hemoglobin measurements.

Generally, hypochromic microcytic anaemia commonly results in low MCHC which was present in 8.2% of participants; nevertheless, their median of MCHC was near normal range. Also, when MCV was below its normal range in about 31.4% of participants, hemoglobin and MCH were below their normal ranges; which suggest, without the possibility of confirmation in this study, the presence of chronic disease and poor iron diet; however, participants did not show anaemic clinical symptoms.

Like our previous study^15^ published in 2016, the present study shows that female students in Syria smoke waterpipe more than cigarette. In fact, between 2014 and 2018 there are several significant changes, such as the expansion of waterpipe smoking from 27.7% to 42.5%, the shrinkage of practicing sport habit from 72.3% to 54.1%, and, the increased percentage of female students who have low hemoglobin from 31.1% to 52.2%. These two percentages are in 95% credibility interval (11% - 56%) of WHO estimation of hemoglobin concentrations <12 g/dL in non-pregnant women aged 15–49 years for the Syrian Arab Republic^35,36^. However, the estimation of WHO was not based on direct measurements of hemoglobin. Nevertheless, considering 11.5 g/dL is the lower limit of hemoglobin normal range in the present study decreases the percentage from 52.2% to 31% which postulates the possibility of a subtle difference in the conditions of measurements between the two studies. Or and after taking into the account that folate levels were normal in the majority of participants and apart from a presumable deterioration of diet patterns, iron intake, and genetic factors; the other possibility could be related to the above mentioned findings, smoking increase and practicing sport decrease, which could be partially associated with hemoglobin concentration decrease. Especially that the positive correlation of hemoglobin along with sport practicing is well known^37^ and it was present in this study.

Folate and other B vitamins are implicated in homocysteine metabolism; hence, Clarke with his colleagues have hypothesized in 2010 that lowering effect of folate and other B vitamins on homocysteine levels can have an impact on cardiovascular disease^38^.

However, Bazzano in his meta-analysis study in 2011 concluded that although homocysteine levels are reduced, there is no effect of folic acid supplementation on cardiovascular events, cancer or mortality after 5 years in people at increased CVD risk^39^.

In contrast, Fatahi and her colleagues preformed a systematic review and meta-analysis of randomized controlled trials in 2018. And suggested that folic acid supplementation could significantly lower the serum CRP level^40^. This study is similar to our present study and included ten studies dated between 2003 and 2016 with a total of 1179 participants. Only 210 participants of them were included in four articles dated between 2014 and 2016. Hence, we will discuss the relation between hsCRP, as CVD biomarker, and folate throughout studies dated between 2006 and 2015 with a total of 805 participants. The only study in common between Fatahi study and our below discussion was Solini study^5^ that included 60 participants. These studies were summarized with the present study (207 participants) in Table 8.

**Table 8:** Latest studies that discussed the effect of folate on hsCRP or the correlation type between them.

Study	Study design: Participants (approximate age mean ± SD years), number of participants in group (number of female) vs. …	Effect^a^ (p value), correlation type (p value)	NR
Solini *et al*.^5^.	Unmasked randomized trial of 3 months: Healthy normal glucose tolerance overweight volunteers (50 ± 7), 30 (19) received folic acid vs. 30 (22) received placebos.	Decrease (0.03)^b^, Inverse (0.04)^c^	No
Chang *et al*.^6^.	Unmasked randomized trial of 3 months: Hemodialysis patients (56 ± 13), 61 (30) received folic acid and vitamin B vs. 60 (30) controls.	Decrease (<0.001)^d^, –	yes
Vayá *et al*.^7^.	Cross-sectional with control: Metabolic syndrome patients (50 ± 10), 61 (20) vs. 98 (39) healthy control.	No ( < 0.001)^e,f^, –	yes
Mierzecki *et al*.^8^.	Unmasked trial of 3 months: Caucasian individuals with atherosclerosis risk factors (28 ± 6), 124 (64) received folic acid.	No (**>**0.05)^e,b,g^, –	yes
Mahalle *et al*.^9^.	Cross-sectional without control: Patients who had coronary artery disease on angiography (60 ± 12), 300 (84).	–, No (0.685)^h^	no
Baszczuk *et al*.^10^.	Unmasked trial of 45 days: Patients with primary arterial hypertension without complications of hypertension and/or coexisting diseases (45 ± 13), 41 (20) received folic acid.	Decrease (**<**0.05)^d^, No (0.578)^i^	no
Present study	Cross-sectional without control: Healthy females participants (21 ± 2), 207 (207).	–, No (0.621)^j^	yes

Note: SD, standard deviation; NR, is the normality of distribution of hsCRP or folate levels was tested? (yes/no) and all above studies did not test the normality of multivariate or bivariate distribution of hsCRP with folate levels; –, not mentioned;

^a^of folate on hsCRP.

^b^Student’s paired t-test of hsCRP levels between two groups.

^c^Multivariate regression analysis considering hsCRP levels as a factor.

^d^Wilcoxon rank sum test of hsCRP levels.

^e^hsCRP values were log transformed to improve normality for statistical analyses.

^f^Student’s unpaired t-test of hsCRP levels between two groups, in contrary, folate levels did not significantly differ (p = 0.977).

^g^Two-way ANOVA post hoc considering dyslipidaemia, BMI and smoking as factors.

^h^Pearson correlation coefficient.

^i^Spearman correlation coefficient.

^j^Kendall’s tau-b correlation coefficients.

While the present study, Vayá *et al*.^7^, Mierzecki *et al*.^8^, and, Mahalle *et al*.^9^ found no effect or correlation between folate and hsCRP; Solini *et al*.^5^, Chang *et al*.^6^, and, Baszczuk *et al*.^10^ showed a decrease in hsCRP levels in participants treated with folic acid. And only Solini *et al*.^5^ showed an inverse correlation between folate and hsCRP.

Further analysis reveals that studies that did not find any effect or correlation between folate and hsCRP had, separately, numbers of female participants bigger than the opposite studies’ numbers, and had cross-sectional design, except the study of Mierzecki *et al*.^8^ which was an unmasked trial, similar to the opposite studies where participants received folic acid.

Among similar studies to Mierzecki’s study design category, Mierzecki’s study was the biggest and its statistical method depended on parametric normal distribution of log transformation of hsCRP. The opposite studies either used parametric methods without testing the normality of the hsCRP distribution or used the non parametric methods.

The present study was the biggest with its 207 female participants and was the only study limited to females. In addition, among its group, it was the only study that used the non parametric statistical methods.

Also, the participants in the present study were the youngest but the range of participant’s age of Mierzecki’s study intersects with the reported in the present study. Hence, unlike the other studies, the participants in these two studies were, approximately, under thirty years old.

The other common point between these two studies was the relatively good health status of participants.

In other studies, participants were around fifty five years old and were diagnosed with one illness at least except participants in Solini *et al*. (2006) and the control group of Vayá *et al*. (2011) studies, who had a relatively good health.

Unfortunately, due to the descriptive design of the study, we could not study the evolution of hsCRP and folate levels, and their effects as risk factors for CVD in our female participants. In addition, homocysteine, iron, vitamin B12, diet and variance in genes involved in folate metabolism were not investigated in this study which limits the interpretation of its results.

Another limitation is that the observed findings were based on a group of female students in a private university, with a fine economical and cultural level, not suffering from diseases that might have influenced the levels of studied hematological and biochemical markers. In addition the age range of participants, 19 to 24 years, presents a difficulty to expand results of present study to larger population.

## Conclusion

In healthy student female adults, this study reported that folate did not correlate with hsCRP. In addition, after reviewing similar works, especially when considering that both Mierzecki’s study^8^ and the present study had participants with comparable health status and age; it is suggested that the possible correlation between hsCRP and folate could be displayed in patients older than 30 years.

Only hsCRP level was significantly higher in high BMI group of participants. Waterpipe smoking, sport practicing, and, participant’s parents’ medical history influenced the correlations of anthropometric and hematological biomarkers measurements along with hsCRP or folate Levels.

Only 3.4% of participants had low folate levels. Meanwhile hsCRP results showed that 20.8% of participants were at average risk of CVD and 2.4% were at high risk of CVD.

The low level of hemoglobin occurrence is increasing in child bearing females, and needs larger and more complete studies that take into account all the possible causes.

Finally, the habit of waterpipe smoking is spreading, and, sport practicing is shrinking. Hence, young females in Syria are advised to consider a healthy lifestyle, free of smoking and packed with physical activity.

## Supplementary information

Supplementary Datatset 1

## Data Availability

All the data used to support the findings of this study are included within the Supplementary Dataset 1.
